# Electronystagmography versus videonystagmography

**DOI:** 10.1590/S1808-86942010000300021

**Published:** 2015-10-20

**Authors:** Maurício Malavasi Ganança, Heloísa Helena Caovilla, Fernando Freitas Ganança

**Affiliations:** 1 Full professor of otorhinolaryngology, Sao Paulo Federal University, Paulista School of Medicine (UNIFESP-EPM). Faculty member of the professionalizing master's degree program in vestibular rehabilitation and social inclusion, Bandeirante University, Sao Paulo (UNIBAN); 2 Associate professor of the otology and otoneurology discipline, Sao Paulo Federal University, Paulista School of Medicine (UNIFESP-EPM); 3 Adjunct professor of the otology and otoneurology discipline, UNIFESP – EPM. Post-doctoral work at UNIFESP – EPM. Collaborating professor of the professionalizing master's degree program in vestibular rehabilitation and social inclusion, UNIBAN. Sao Paulo Federal University, Paulista School of Medicine. Bandeirante University, Sao Paulo

**Keywords:** electronystagmography, nystagmus, pathologic, eye movements

## Abstract

Electronystagmography (ENG) and videonystagmography (VNG) are eye movement recording methods used for the evaluation of balance disorders.

**Aim:** To compare literature information on the similarities, differences, advantages e disadvantages between ENG and VNG.

**Materials and Methods:** review of the scientific literature.

**Results:** ENG and VNG are very helpful methods for evaluating balance disorders, due to their capacity to recognize signs of peripheral or central vestibular dysfunction and to pinpoint the side of the lesion. Major advantages of VNG are related to calibration, temporospatial resolution, and recording of horizontal, vertical and torsional eye movements.

**Conclusion:** VNG is a new technology that presents advantages in the evaluation of eye movements; however, despite its disadvantages, ENG is still considered a valuable test in the clinical setting.

## INTRODUCTION

Laboratory functional vestibulo-oculomotor testing should be done in patients with vertigo and other types of dizziness or unbalance with suspected vestibular dysfunction.^1^, ^2^, ^3^, ^4^ Vestibular testing is an addition to the clinical history and physical examination, and supports the diagnosis and treatment of vestibular diseases.^1^, ^2^ It should be noted that the diagnosis of vestibular diseases is not based only on the results of vestibular function tests, and that the patient's disability does not correlate with the degree of functional loss that is found on these tests.^5^

Altered findings suggest signs of peripheral or central vestibular dysfunction and may establish the affected side.^2^, ^3^, ^6^, ^7^, ^8^ Abnormal findings should be duly characterized, relevant findings should be valued, and the results should be interpreted jointly; the type and intensity of vestibular involvement may help define the prognosis.^6^

The routine assessment of patients with dizziness and bodily balance disorders includes recording and analyzing the types of nystagmus and other eye movements.^3^,^8^ It is preferable to record – rather than merely observe – nystagmus, as abnormal movements and the effect of visual fixation may be recognized and quantified, and successive tests may be stored, recovered and compared.^3^ Recording makes it possible to measure quantitative parameters such as the speed of the slow component in different types of nystagmus, latency, saccade accuracy and velocity, and gain in ocular tracking and optokinetic nystagmus.^6^

There is no consensus about which procedures should be included in an otoneurological test battery; the most commonly used are test for investigating positioning and positional nystagmus, spontaneous nystagmus, semi-spontaneous nystagmus, saccades, ocular tracking, optokinetic nystagmus, per- or post-rotatory nystagmus, and post-caloric nystagmus.^2^, ^3^, ^6^, ^7^, ^8^

Electronystagmography or eletro-oculography (ENG) is the most commonly used method for recording eye movements. It applies the corneal-retinal potential variation principle during eye movements to record and analyze functional features of the vestibulocochlear reflex, and of the saccadic, pursuit, optokinetic and fixation visual systems.^2^, ^3^

Videonystagmography or video-oculography (VNG) is a computerized system that applied the principle of recording eye movements by using infrared sensors in special spectacles or masks. The computer software measures and analyzes eye movements, which may be presented on a video monitor and recorded.^6^

The reason for presenting this review of ENG and VNG is the paucity of papers in our context that compare both procedures.

The purpose of this paper was to present the similarities, differences, advantages and disadvantages of ENG and VNG for investigating vestibulo-oculomotor function, based on the pertinent scientific literature.

## METHOD

A review of information about ENG and VNG was carried out in April 2009 in the MEDLINE electronic database and in published books on otoneurology, among others. The database search strategy included the keywords: “electronystagmography” or “videonystagmography” or “video-nystagmography” or “video nystagmography” or “video-oculography” or “video oculography” or “videooculography” or “electronystagmography and videonystagmography.” The filter “humans” and the languages English, Portuguese, French and Spanish defined the scope of the search.

Resulting titles and/or abstracts were analyzed according to the inclusion criteria for the review: the technical features of eye movement recording methods (eletronystagmography, vector-electronystagmography and videonystagmography), their advantages and disadvantages, and comparisons between these methods. Studies describing these methods for the evaluation of otoneurological diseases only were excluded. A critical review was made of the content.

## REVIEW OF THE LITERATURE

Our keyword-based search yielded 2,233 references. We read the titles and found 60 that could be assessed based on our criteria. Detailed reading excluded 25 papers that did not meet the inclusion criteria. Thus, 35 sources about the theme met the inclusion criteria of this review and were selected for a critical content analysis.

ENG is an eye movement recording procedure where the eye acts like a battery: the cornea is the positive pole and the retina is the negative pole. Electrodes located in specific periorbitary points pick up the corneal-retinal electrical potential variation caused by eye movements, which are then amplified and sent to the recording device.^1^, ^2^, ^3^

ENG devices generally have two or more recording channels. It is possible to simultaneously record horizontal and vertical movements (one channel each) with eyes open or closed in two-channel devices. There are several ways to place the electrodes; this depends on what is to be studied and the number of available channels. A reference electrode (ground) is placed on the frontal medial line. Horizontal movements in both eyes may be detected in one channel (horizontal) by an active electrode placed on the skin next to the right and left external periorbitary corners. The purpose of the other channel (vertical) is to detect vertical movements in each eye by a second pair of electrodes, one above and one below the eye. Each eye may be assessed separately; in this case, one recording channel is used for detecting horizontal right eye movements and the other channel for detecting horizontal left eye movements. Each of these channels is configured by placing an active electrode next to the external corner and another next to the internal corner of the eye. Four channels make it possible to record horizontal and vertical eye movements separately for each eye.^2^, ^3^,^6^

The convention for the horizontal channel is to place the electrodes such that a displacement to the right of the gaze corresponds to an upward eye movement on the recording device, and when the gaze is displaced to the left, the device will record a downward movement. The convention when placing electrodes for the vertical channel is that an upward displacement of the gaze corresponds to an upward eye movement recording, and a downward displacement of the gaze corresponds to a downward eye movement recording. Adequate gauging of eye movements is needed to read the recordings correctly: the ocular deviation angle should be represented by specific recorded movement amplitudes on the tracing. Gauging makes it possible to read and interpret the tests under similar conditions. Recalibration is recommended along the test.^3^

The diagnostic power of ENG increased with the advent of computerized technology.^6^,^9^, ^10^, ^11^ ENG is a simple, non-invasive and reasonably accurate procedure for routine vestibular assessment; there are, however, some disadvantages, such as interference from muscle electrical activity and ambient electric noise.^9^ The corneal-retinal potential, which is used for indirectly measuring the eye position across about 40° horizontally and vertically, varies among people and may be absent. It changes according to light and while the test is being done (even without changes in lighting), which requires several calibrations. The eye movement baseline recording may deviate; resolution is poor (about 1°) and does not separate eye movements below 2° to 3°; tracing quality depends on the intensity of the corneal-retinal potential; blinking or electromyographic interference may contaminate vertical eye movement recordings; and ENG does not measure or record torsional eye movements.^9^,^12^, ^13^, ^14^, ^15^ ENG may not reliably be used for eye movements less than 5°.^16^

It is not possible in ENG to adequately interpret the findings of the Dix-Hallpike test in cases of benign paroxystic positional vertigo (BPPV), as the torsional component of positional and positioning nystagmus is not recorded, and recording the vertical component is subject to artifacts and contamination by blinking.^13^, ^14^,^17^

ENG helped identify peripheral vestibular dysfunction in 45.1% of cases, or central vestibular dysfunction in 6.9% of cases. Magnetic resonance imaging provided the topographical diagnosis in only 3.9% of cases among 102 patients with vertigo and location instability of poorly defined lesions in the clinical history.^18^ ENG decreased the percentage of unclear diagnoses from 34.2 to 13.8% when the diagnosis was unclear in the clinical history and audiological, neurological and laboratory testing.^19^A clinician would not identify labyrinth disease in 66.0% of cases if ENG were used only for patients that reported vertigo; this error could become a significant legal liability for physicians.^7^

Vector-eletronystagmography (VENG) is a variant of ENG that uses three channels to record eye movements. VENG also starts with picking up corneal-retinal electric potential variations upon eye movements. An active electrode is placed next to the external corner of each eye and the third electrode is placed on the frontal midline in such a way that the three recording channels are configured as an isosceles triangle. Three bipolar derivations are set from the active electrodes, thereby making it possible to identify horizontal, vertical and oblique eye movements. Measuring the slow component velocity of nystagmus takes into account the directional influence of responses according to the vector projection of eye movements. The horizontal channel in VENG is similar to the horizontal channel in ENG. VENG makes it possible to investigate oblique nystagmus resulting from vertical semicircular canal stimulation in rotatory testing by tilting the patient's head 60°$ backwards and 45° sideways.^6^,^20^, ^21^, ^22^, ^23^, ^24^, ^25^

Eye movement tracings based on videotechnology have become popular because of rapid developments in electronic data processing, affordable devices, more robust algorithms, and a wider range of uses.^16^

VNG is a computerized method that does not use electrodes.^6^ It employs a source of invisible infrared light and can record eye movements under any conditions of ambient light, even in darkness.^9^ Video cameras installed in light-proof binocular lenses have made it possible to directly observe and record horizontal, vertical and torsional eye movements with eyes open and in darkness. VNG employs digital image processing to measure movements from the center of the pupils; it is also possible to measure the slow component velocity of horizontal and vertical nystagmus.^6^,^26^, ^27^ VNG spectacles should be firmly placed over the patient's head, as camera movements relative to the head will yield eye movement recording artifacts;^28^ a one millimeter translation will cause an about 5° error.^16^

VNG assesses eye position over about 30° horizontally and about 20° vertically with a resolution of about 0.1°; it may detect eye movements of 0.5° in laboratory conditions. The vertical and horizontal channels have a similar resolution in VNG. Eye images may be digitally recorded in computers at the same time as the tracings. Tracing quality depends on image quality. Gauging depends on the distance between the eye and the camera. After initial gauging, repeat gauging is only needed if the spectacles or the camera are repositioned.^13^, ^14^ It is possible to visualize, but not to measure, torsional eye movements with VNG.^5^,^13^, ^14^

One of the main advantages of VNG is to visualize and record the exact direction of eye movements. VNG is useful for assessing positioning and positional nystagmus, and especially for the diagnosis of BPPV, which is one of the most common vestibular diseases.^6^,^13^, ^14^,^17^ Nystagmus in the Dix-Hallpike test may be of low amplitude, short duration and not always identified or characterized upon visual observation.^6^ Monitoring with VNG is useful for the diagnosis and recording of eye movements during therapeutic maneuvers in BPPV.^29^ VNG is a functional, differential diagnostic, and monitoring test for several conditions.^30^

VNG tracings are clean and do not deviate from the baseline of eye movements; the analysis and interpretation are therefore more accurate. The procedure is easy to perform and faster than when electrodes are used; gauging is needed only at the beginning. The equipment is more costly, and some claustrophobic patients do not tolerate the feeling of confinement. Patients with palpebral ptosis or eyelashes that cover the pupils may be hard to assess with VNG.^26^ Cosmetics around the eyes may interfere with infrared light and affect the assessment.^27^ VNG may be difficult to perform in children below the age 5 years, since they may not tolerate the mask because of their small faces. Patients with diseases that affect the shape of the pupil and patients that for any reason are unable to keep their eyes open are also harder to evaluate with this method. Degenerative, diabetic or hypertensive retinopathies, and retinitis pigmentosa may alter the magnitude of the corneal-retinal potential.^31^

ENG and VNG are valuable procedures in patients with vertigo. The practical side is that both methods are useful; indicating ENG or VNG depends on the their limitations in each case. ENG is the test of choice in about 1% of cases in which VNG cannot be done; it is therefore recommended to have both tests available.^14^ The usefulness of recording eye movements depends highly on the technical knowledge and training of the professionals that carry out and interpret these tests; this may cause significant variation in results among laboratories.^4^

VNG has gradually replaced ENG in recent years.^30^,^32^Both methods, however, are more clinical valuable than any other laboratory test for assessing the vestibular system; lesions may be detected, peripheral and central conditions may be differentiated, and the side of a lesion may be established. ENG and VNG detect one or more alterations in about 50% of patients with dizziness, and specify the site in about 75% of these abnormalities. Other laboratory tests detect few of these conditions. An experienced physician may detect most of these abnormalities in the physical examination, but cannot provide a quantitative analysis or a permanent record. ENG and VNG help define treatment strategies, monitor the progression of therapy, and to plan surgery for vestibular schwannomas, vestibular ablation and cochlear implants.^32^

ENG and VNG are well-tolerated non-invasive laboratory tests to measure and record eye movements. ENG is not recommended for blind patients or those with poor corneal-retinal potentials, and cannot detect very small eye movements; these limitations may be overcome by observing the eyes directly or in video recordings.^8^ VNG uses two video cameras to film the eyes (monocular or binocular recording); recording is only possible with the eyes wide open. Computer analysis is done to present eye movements in two dimensions. Three-dimensional VNG for added measures of torsional eye movements requires extensive image analysis of the iris or sclerotic marking points; this is still a complicated and expensive procedure.^15^ VNG has many advantages over conventional ENG. It is performed easily and rapidly, saccade velocity calculations are more accurate (this method uses a 240 Hz scanning speed), there are no artifacts of muscular origin, it records without filtering low frequencies, it eliminates electrical interference, recordings are stable with time, it can assess eye movements under any light conditions (spectacles make it possible to record in the dark, torsional eye movements may be observed directly, and visualized eye moments correlate with the tracings.^9^ ENG is more affordable than VNG.^29^ ENG is used in cases that require measures of eye movements with eyes closed; it is the only method that offers this option.^16^

Differences between ENG and VNG include gauging, time and space resolution, and the specificities of the eye movement plane.^33^ Although ENG has disadvantages of electrical artifacts, longer duration of the procedure, and variable gauging, it is a less costly, reliable and accurate measure of the slow component of eye movements.^8^ An advantage of VNG is the possibility of visually reviewing eye movements to clarify doubts. A caveat of this method is that claustrophobia may make it intolerable for patients to use the mask; in these cases, ENG may be used.^34^ In general, both procedures are useful^34^, ^35^ and convenient for patients and examiners.^35^

## FINAL COMMENTS

Assessments of vestibular function are not specifically designed for diagnosing diseases or their etiology; these depend on the patient's clinical history and ensuing exams. A comparison of results with the clinical history yields clinical meaning to the findings of vestibular studies.

The literature shows that ENG and VNG are useful for the diagnosis of body balance disorders; these tests may provide information about the existence of altered central or peripheral vestibular function and define the side of the lesion. ENG and VNG results add to the clinical history, the otorhinolaryngological evaluation, and other tests in patients with vertigo and other types of dizziness and/or unbalance. The literature suggests that VNG is more technologically advanced and advantageous compared to ENG; but adds that ENG remains a semiologically valid procedure, such as when VNG cannot be done.
